# Blue-violet light decreases VEGFa production in an *in vitro* model of AMD

**DOI:** 10.1371/journal.pone.0223839

**Published:** 2019-10-23

**Authors:** Mélanie Marie, Pauline Gondouin, Delphine Pagan, Coralie Barrau, Thierry Villette, José Sahel, Serge Picaud

**Affiliations:** 1 Sorbonne Université, INSERM, CNRS, Institut de la Vision, Paris, France; 2 Essilor International R&D, Charenton-le-Pont, France; 3 Department of Ophthalmology, The University of Pittsburgh School of Medicine, Pittsburgh, PA, United States of America; Eye Hospital, Charité, GERMANY

## Abstract

Blue light is an identified risk factor for age-related macular degeneration (AMD). The production of vascular endothelial growth factor (VEGF), leading to neovascularization, is a major complication of the wet form of this disease. We investigated how blue light affects VEGF expression and secretion using A2E-loaded retinal pigment epithelium (RPE) cells, a cell model of AMD. Incubation of RPE cells with A2E resulted in a significant increase in VEGF mRNA and, intracellular and secreted VEGF protein levels, but not mRNA levels of VEGFR1 or VEGFR2. Blue light exposure of A2E-loaded RPE cells resulted in a decrease in VEGF mRNA and protein levels, but an increase in VEGFR1 levels. The toxicity of 440 nm light on A2E-loaded RPE cells was enhanced by VEGF supplementation. Our results suggest that age-related A2E accumulation may result in VEGF synthesis and release. This synthesis of VEGF, which enhances blue light toxicity for the RPE cells, is itself suppressed by blue light. Anti-VEGF therapy may therefore improve RPE survival in AMD.

## Introduction

Age related macular degeneration (AMD) is characterized by a loss of central high acuity vision [1]. The dry type of AMD is the most common, accounting for approximately 90% of patients and is characterized by retinal pigment epithelium (RPE) and photoreceptors degeneration over time in the macular area [2]. The wet type affects approximately 10% of patients and is characterized by abnormal blood-vessel growth [2]. Such blood-vessel growth is mainly attributed to the release of vascular endothelial growth factor (VEGF), as shown by the success of anti-VEGF therapies [3, 4]. Anti-VEGF therapies have become a major treatment modality in the daily care of wet AMD to suppress the growth of neovessels, which invade the retina from the underlying choroid, located below the retinal pigment epithelium (RPE) [3, 5]. VEGF traps have proven to be highly effective in protecting or even restoring visual function. VEGF, which is secreted by the retinal pigment epithelium [4], is indeed a very potent angiogenic factor in the retina. Although VEGFa binds to both VEGF receptors VEGFR1 (Flt-1) and VEGFR2 (KDR/Flk-1), VEGFR2 appears to mediate almost all intracellular signaling pathways in the vascular endothelium [6, 7]. Understanding the molecular mechanisms that regulate VEGF production is critical for preventing and controlling the development of this major complication of AMD.

Several risk factors for the development of AMD have already been identified, including age, smoking, genetic factors, and exposure to sunlight [2, 8, 9]. The role of sunlight exposure has been demonstrated in various epidemiological studies [2, 10–15] and is corroborated by the protective role of macular pigments [16, 17]. Indeed, within the solar spectrum, blue light is more highly involved in disease induction, consistent with the blue-light filtering capacity of macular pigments [13, 18–23]. This severe retinal disease is characterized by the accumulation of lipofuscin within the RPE [24]. RPE cells ultimately degenerate in parallel with the loss of photoreceptors [1]. A2E is one of the retinoid compounds of lipofuscin which can enhance VEGF expression [25, 26] and cause RPE degeneration, even in darkness [27]. At low concentrations, A2E can act as a photosensitizer to induce cellular apoptosis upon blue-light exposure [28–38]. Controversial results on the effect of light on VEGF expression have been reported. In the human ARPE-19 cell line, acute white light (2500 lux for 12 h or 10 mW/cm^2^ for 30 min) or short blue light (430 nm, 1 mW/cm^2^, for 3 to 7 min) exposure have been shown to enhance VEGF expression [39–41]. In addition, Kernt *et al*. confirmed that primary human RPE cells containing lipofuscin exhibited increased VEGFa mRNA levels when exposed to white light for 15–60 min at 350 mW/cm^2^ [42, 43]. In contrast, white light (5000 lux 1 h) was shown to downregulate VEGF in the RPE-choroid eye cup, while increasing its expression in the neural retina of albino mice [44].

We recently demonstrated that blue light (415–455 nm) of the solar spectrum reaching the retina is the most toxic spectral band for A2E-loaded primary porcine RPE cells, used as an *in vitro* model of AMD [45, 46]. Here, we examined how blue light affects VEGF levels in AMD. We used this *in vitro* model to investigate how blue light affects VEGF mRNA and protein levels and whether VEGF modifies RPE cell survival via the VEGFR2 pathway.

## Materials and methods

### Cell culture

Porcine eyes were bought at a local slaughterhouse (Etablissements guy Harang, Houdan, France) in agreement with the local regulatory authorities and the slaughterhouse veterinarians (agreement FR75105131). This procedure adheres to the European initiative for restricting animal experimentation because not a single animal was killed for our experimentation. Eyes were taken from animals slaughtered daily for food production. Retinal pigment epithelium cells (RPE cells) were extracted as previously described [45]. Three days after seeding in 96-well plates, confluent cells were treated for 6 h with 0, 12.5, or 20 μM A2E (Orga-Link, Magny-les-Hameaux, France) in DMEM (Dulbecco's Modified Eagle Medium, Life Technologies, Carlsbad, CA, USA) without serum. DMSO (Sigma-Aldrich, St Louis, MO, USA) was adjusted to a final concentration of 0.1% for all conditions. After A2E treatment, cells were washed twice with modified DMEM (medium without any photosensitizer, such as phenol red, riboflavin, folic acid, or aromatic amino acids; Life Technologies) and exposed to light.

### VEGF supplementation

In experiments designed to investigate the effects of exogenous VEGF on cell viability and apoptosis, 10 ng/mL recombinant VEGF (R&D System, Minneapolis, MN, USA) was diluted in modified DMEM and added to the cells 2 h before light exposure. Control cells were treated with the same concentration of the VEGF diluent alone (PBS-0.1% BSA, Sigma-Aldrich). Viability and apoptosis were assessed 6 h after the end of light exposure using the ApoLive-Glo^™^ Multiplex Assay (Promega, Madison, WI, USA) as previously described [45].

### Inhibition of VEGF signaling

The effects of the inhibition of VEGF signaling on cell viability and apoptosis were investigated using a selective inhibitor of VEGFR2 (ZM323881 hydrochloride, Tocris, Bristol, UK) diluted to 1 μM in modified DMEM. Control cells were treated with the same concentration of the VEGFR2 inhibitor diluent alone (DMSO). Cells were treated just before light exposure. Viability and apoptosis were assessed 6 h after the end of light exposure using the ApoLive-Glo^™^ Multiplex Assay (Promega) as previously described [45].

### Light conditions

Cells were exposed to 10 nm-bandwidths of light produced by a specific LED-based optic fibers illumination device as previously described [45]. Physiological light conditions on the retina were mimicked by exposing RPE cells to a normalized light spectrum obtained by applying the ocular media filtering spectrum onto a referenced solar spectrum (ASTM G173-03, International standard ISO 9845–1, 1992); blue light is partially filtered by the anterior ocular media as a natural protector. Irradiances ranged from 0.11 at 400 nm to a maximum irradiance level of 1.5 mW/cm^2^ obtained for the light band centered at 630 nm [45, 46]. Irradiance level, spectral, and uniformity measurements were obtained using a calibrated JAZ spectroradiometer (Ocean Optics, Dunedin, USA). For qPCR experiments, cells were exposed to light for 15 h and directly characterized or maintained in darkness for 6 to 24 h prior to characterization. For ELISA experiments cells were directly characterized after light exposure. For viability and apoptosis measurements, cells were exposed to light for 18 h and maintained in darkness for 6 h as previously described [45].

### Enzyme-linked solid phase immunosorbent assay

The Quantikine Human VEGFa Immunoassay (R&D Systems) was used to quantify VEGFa protein levels in cells and the cell culture medium. After 15 h of light exposure, media was collected, centrifuged to eliminate cell debris, and the supernatants collected and stored at -80°C. Cells were then rinsed twice with PBS (Life Technologies) and incubated for 5 min on ice in cell lysis buffer (Cell Signaling Technologies, Danvers, MA, USA) containing an antiprotease cocktail (Roche, Indianapolis, IN, USA). Cell lysates were sonicated, centrifuged, and the supernatants collected and stored at -80°C. VEGFa protein content in cell lysates and cell culture media was measured by ELISA according to the manufacturer’s instructions. Total protein content was determined for cell lysates using the micro BCA Protein Assay Kit (Thermo Scientific, Rockford, IL, USA).

### Real-time PCR

At the end of light exposure or after a 6 to 24 h rest period in darkness, cells were collected and total RNA isolated using the RNeasy Micro Kit (Qiagen, Hilden, Germany). RNA quality and quantity were assessed by spectrophotometry (NanoDrop 2000, Thermo Scientific). cDNA synthesis was performed using SuperScript II (Invitrogen) with random primers (Promega), 10 mM dNTPs (Life Technologies), and 0.1 M DTT (Life Technologies). The mix was incubated for 10 min at room temperature, then 50 min at 42°C, and finally 15 min at 70°C. Real-time PCR was performed using a StepOne device (Life Technologies) in a mixture containing cDNA, SYBR Green master mix (Life Technologies) and VEGFa forward (5’CACAGGACGGCTTGAAGATG3’) and reverse (5’TCTACCTCCACCATGCCAAG3’) primers, or VEGFR1 forward (5’CTCAACGCCATTCTGACGAG3’) and reverse (5’GGCGTTTGGGGAAAGTTCTT3’) primers, or VEGFR2 forward (5’CAAGAGGATGTTTCGAGCCG3’) and reverse (5’AAATCCCTCAGCGATGTGGA3’) primers. Data were normalized to that of 18S RNA, which was simultaneously amplified for all samples using 18S forward (5’AGTCGGCATCGTTTATGGTC3’) and reverse (5’CGCGGTTCTATTTTGTTGGT3’) primers. A total of 1 ng of cDNA was sufficient to correctly amplify the VEGF gene, whereas 5 ng of cDNA was necessary for VEGFR1 and VEGFR2. Indeed, VEGFR2 was barely detectable after 6 and 24 h post light exposure, even when using 15 ng of cDNA, and the data could not be properly interpreted because of the low quantity of final amplification products. RT-PCR amplification products were purified and sequenced using the Sanger method and the sequences compared to those of the pig genome. mRNA level ratios were determined using the delta Ct calculation method: Δ*C_T_* = *C_T target gene_*−*C*_*T* 18*S*_ and ΔΔ*C_T_* = Δ*C_T sample_*−Δ*C*_*T dark control* 0 μ*M*_, relative mRNA level to dark control 0 μM = $2^{-\Delta \Delta C_{T}}$.

### Statistical analysis

All experiments were repeated at least three times. Data are presented as the mean +/- SEM. Statistical analyses were performed using Statistica software v12 (StatSoft, Tulsa, OK, USA). Two-way ANOVA with repeated measures was used. Bilateral Dunnett post-hoc tests were used to compare variances of all tested groups (for each A2E concentration and each light condition) to the dark control groups. Tukey post-hoc tests were used for cell viability and apoptosis analyses to compare variances of all groups together. Differences between samples were considered to be significant when p < 0.05 (*), p < 0.01 (**), or p < 0.001 (***).

## Results

### Blue-violet light decrease VEGFa mRNA expression and increase VEGFR1 mRNA expression

**Fig 1:**
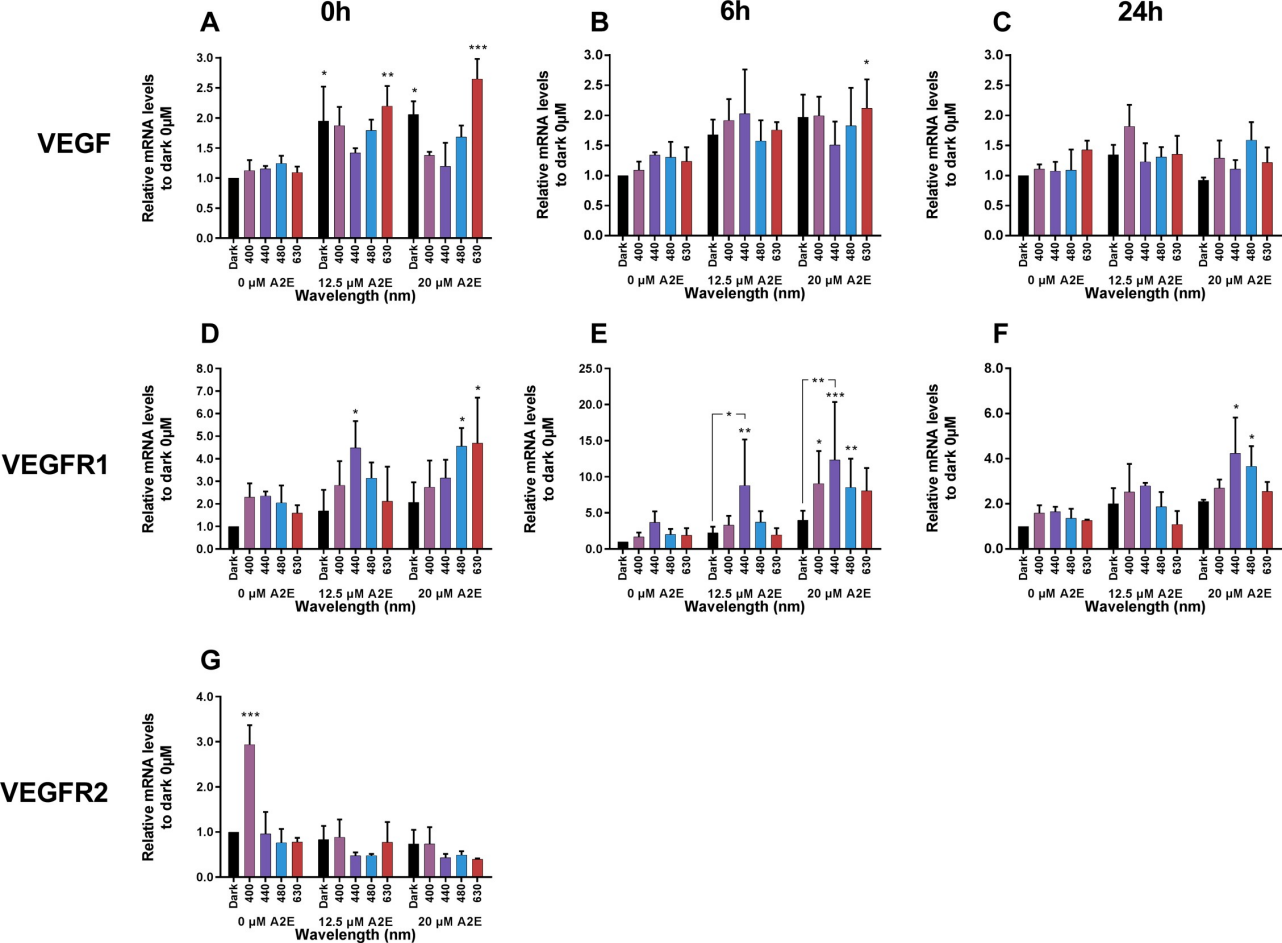
VEGFa, VEGFR1 and VEGFR2 mRNA levels following A2E incubation and/or light exposure. Each histogram represents the mRNA levels of the gene in darkness or following exposure to wavelengths (400, 440, 480 or, 630 nm) in the absence or presence of A2E preloaded at 12.5 or 20 μM. These measurements were repeated for three time points: immediately, 6 h, or 24 h after light exposure. Note the increase in gene expression for VEGF and VEGFR1 after A2E incubation in cells maintained in darkness. Blue light exposure at 440 and 480 nm suppressed the increase in VEGF expression, whereas it further increased VEGFR1 expression. Data are expressed as the mean +/- SEM (n = 3). Differences between experimental samples and 0, 12.5, or 20 μM dark controls were considered to be significant when p < 0.05 (*), p < 0.01 (**) or p < 0.001 (***).

We evaluated VEGFa, VEGFR1 and VEGFR2 mRNA levels by qRT-PCR following A2E incubation and/or light exposure (Fig 1). All mRNA levels were normalized against the level of the 18S ribosomal subunit mRNA, which was not expected to change during the procedures. RPE cells maintained in darkness showed high levels of VEGFa mRNA relative to those of its receptors, VEGFR1 and 2. The average expression ratio to VEGFa reached for VEGFR1 1/142 and 1/1,969 for VEGFR2 (mean delta Ct_VEGF_ = 14.24, mean delta Ct_VEGFR1_ = 21.39 and mean delta Ct_VEGFR2_ = 25.18), in accordance with the results of a previous study [47]. A2E treatment increased the expression of VEGFa and VEGFR1. VEGFa mRNA levels increased by two-fold following incubation of RPE cells with A2E, as previously reported by others [26, 48], although this increase disappeared by 39 h after the end of A2E incubation (Fig 1A, 1B and 1C). Similarly, VEGFR1 mRNA levels also increased up to four-fold after incubation with A2E, and this increase was stable over time (Fig 1D, 1E and 1F). In contrast, VEGFR2 mRNA levels remained very low, even in the presence of A2E (Fig 1G).

We then investigated how light affects VEGFa and VEGF receptor expression in RPE cells by exposing them to various wavelengths (400, 440, 480 and 630 nm) after A2E incubation. RPE cells were exposed for 15 h, as we recently found that this exposure period enabled us to investigate the molecular mechanisms prior to apoptosis [46] by limiting light toxicity observed with longer light exposures (18 h) [45]. We measured VEGFa and VEGF receptor mRNA levels in RPE cells immediately after light exposure and at two additional time points to investigate the long-term effect of light exposures. Light exposure alone did not modify VEGFa mRNA levels in the absence of A2E at any of the post-exposure time points (Fig 1A, 1B and 1C). In contrast, blue light at 400, 440, or 480 nm decreased the A2E-elicited increase in VEGFa mRNA levels, with the largest reduction at the highest A2E concentration (20 μM), reaching 50% at 440 nm immediately after the end of light exposure (Fig 1A, 1B and 1C). VEGFa levels thus returned to the control values measured in the absence of A2E. At later time points after light exposure, differences from the control condition in darkness were steadily disappeared, as the A2E-elicited increase of VEGF expression was also fading away.

We observed a statistically significant increase in VEGFR1 mRNA levels immediately after light exposure at 440, 480, and 630 nm in the presence of 12.5 and 20 μM A2E (Fig 1D). We observed the greatest increase of VEGFR1 mRNA levels 6 h after the end of light exposure at 440 nm, reaching a maximum of 8.8-fold for 12.5 μM A2E and 12.3-fold for 20 μM A2E (Fig 1E). This increase completely disappeared by 24 h after the end of light exposure for 12.5 μM A2E, but VEGFR1 mRNA levels remained elevated relative to dark controls for 20 μM A2E, but at lower levels (Fig 1F). The increase in VEGFR1 expression demonstrates that the decrease of VEGFa mRNA expression is not only due to the reduced number of viable cells.

VEGFR2 mRNA levels only increased in the absence of A2E at the 400-nm wavelength exposure but this effect was not maintained in the presence of A2E (Fig 1G). Incubation with A2E resulted in a decrease of VEGFR2 mRNA levels under blue light (440 and 480 nm), but only at the lowest A2E concentration (12.5 μM) (Fig 1G). Data from later time points were not used due to the very low levels of VEGFR2 mRNA. The gene expression data for VEGFR2 must be interpreted with caution, as VEGFR2 expression was barely detectable, even after increasing the amount of starting material.

### Blue-violet light decreases the intracellular concentration of VEGFa

**Fig 2:**
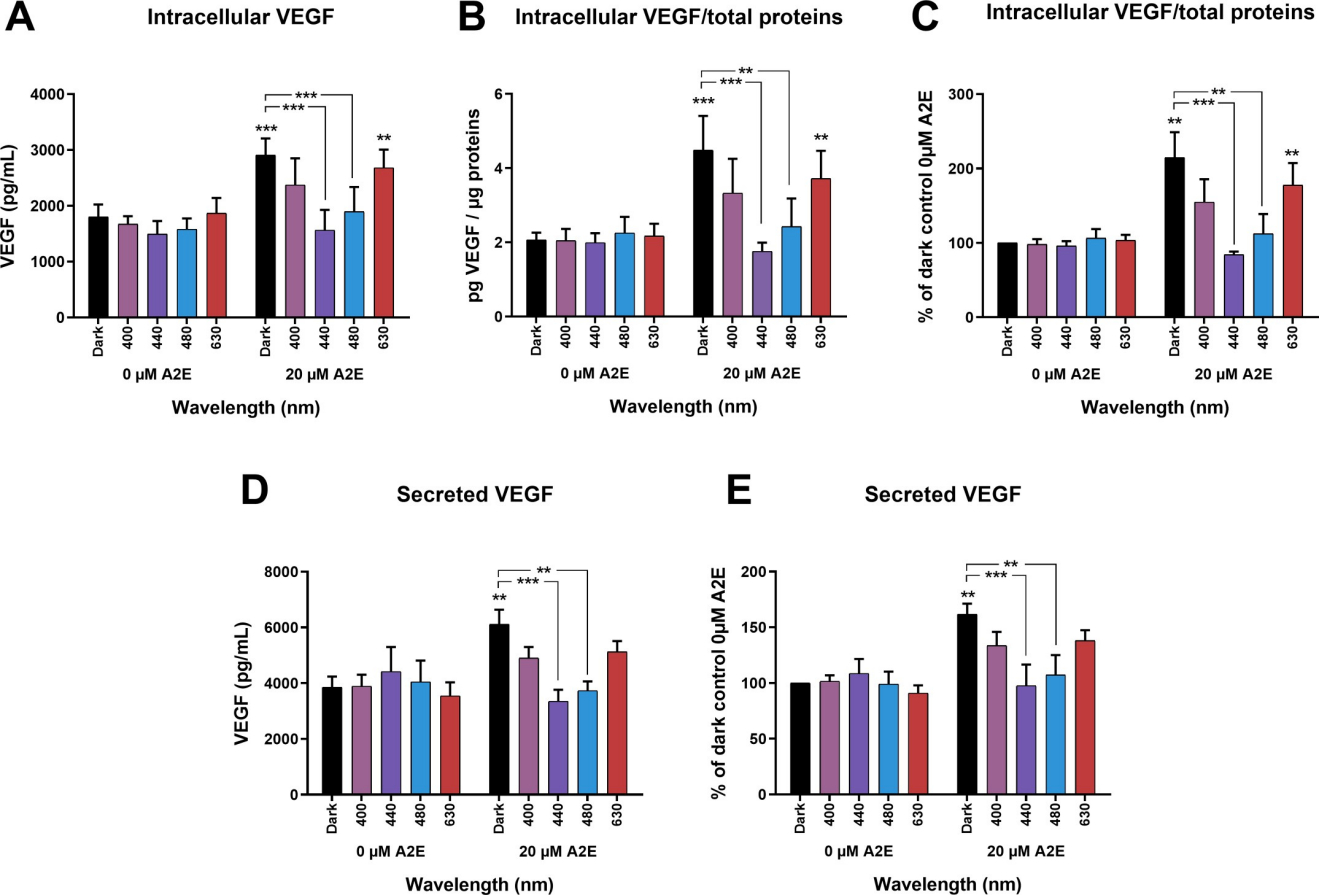
Blue-violet light suppression of the A2E-elicited increase in intracellular and secreted VEGFa protein levels. VEGFa protein contents in RPE cell lysates (A), after their normalization for each sample to its protein content (B), and after a second normalization with respect to the value measured in the dark control without A2E (C). VEGFa protein levels released in the culture media (D), and after their normalization with respect to the value measured in the dark control without A2E (E). Note that A2E increased both the levels of VEGFa in the cells and the secreted VEGFa whereas blue-violet light (440 nm) suppressed these increases in VEGFa proteins levels. These protein levels were measured by ELISA following 15 h of light exposure with or without pre-incubation in 20 μM A2E. Data are expressed as mean +/- SEM (n = 4) for intracellular content and as mean +/- SEM (n = 8) for secreted VEGFa. Differences between experimental samples were considered to be significant when p < 0.05 (*), p < 0.01 (**) or p < 0.001 (***). Stars on the 20 μM columns represent significant differences with the corresponding 0 μM averaged value.

After observing modulation of VEGFa mRNA levels by A2E and light exposure, we examined whether VEGFa protein synthesis was also modified. We evaluated VEGFa protein concentrations in cell lysates by ELISA immediately after light exposure (Fig 2). VEGFa protein concentrations varied between 1.76 and 4.49 pg/μg proteins for all conditions tested (Fig 2B). Light alone did not affect the intracellular content of VEGFa protein in the absence of A2E at any of the tested wavelengths (Fig 2A, 2B and 2C). Incubation of RPE cells with A2E (20 μM) induced a two-fold increase in the intracellular content of VEGFa protein, consistent with the previously observed increase in VEGFa mRNA levels. Light exposure (440, 480 nm) decreased VEGFa protein levels, except for the illumination bands at 400 and 630 nm, in which VEGFa protein concentrations were in the same range as those measured in A2E-loaded cells maintained in darkness. As a consequence, VEGFa protein concentrations returned to levels measured in the absence of A2E treatment following blue light exposure (440 or 480 nm). Thus, blue light highly counteracts the increase in intracellular VEGFa protein content triggered by A2E incubation.

### Blue-violet light decreased VEGFa protein secretion

We next investigated the release of VEGFa protein by RPE cells upon light exposure by measuring its concentration in the cell-culture medium by ELISA at the end of light exposure. Cells were washed before light exposure with fresh VEGF-free medium containing neither serum nor added growth factors. Thus, the measured VEGFa protein concentration in the culture medium corresponded to the protein released during the exposure of RPE cells to light. VEGFa protein concentrations varied from 3,347 to 6,107 pg/mL for all conditions tested. RPE cells maintained in darkness for 15 h secreted a high amount of VEGFa, approximately 4,000 pg/mL (Fig 2D). Light exposure alone did not modify VEGFa protein concentrations in the extracellular medium in the absence of A2E (Fig 2D and 2E). In contrast, incubation with A2E significantly increased VEGFa protein release, consistent with the enhanced intracellular levels of VEGFa in A2E-loaded cells. Again, blue light (440 and 480 nm) significantly reduced the release of VEGFa protein into the cell-culture medium, which returned to the levels measured in the absence of A2E (Fig 2D and 2E). The increase of VEGFa protein levels upon A2E incubation was statistically significant, as well as its suppression by blue light (440 and 480 nm) (Fig 2D and 2E). These results highlight the association between intracellular VEGFa protein content and VEGFa protein release, suggesting that reduced VEGFa protein release is related to the reduced protein synthesis under blue light stimulation. Finally, our measurements at the mRNA and protein levels were consistent, indicating that the observed changes in protein secretion were directly related to regulation of the mRNA levels, which were increased by A2E in darkness and suppressed by blue-violet light. Unlike for the normalized mRNA levels, it cannot be fully excluded that the reduction in VEGF protein levels was not at least partially due to reduced cell viability.

### Effects of VEGFa on cell viability and apoptosis in A2E-loaded RPE cells exposed to blue-violet light

**Fig 3:**
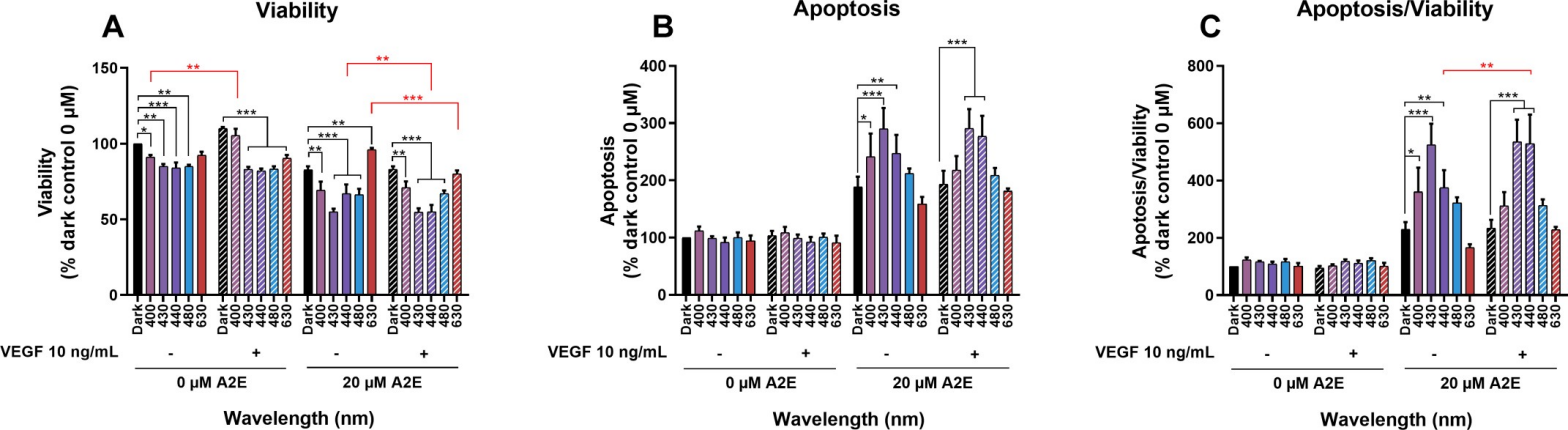
Effects of VEGFa supplementation on cell viability and apoptosis in A2E-loaded RPE cells exposed to light. RPE cells were loaded with 0 or 20 μM A2E and then the media supplemented with 10 ng/mL recombinant VEGFa before being exposed to light for 18 h. Viability (A) and apoptosis (B) were assessed with the Apolive Glo AssayTM after light exposure following a 6 h rest period in darkness. The number of apoptotic cells was normalized to cell viability to define the density of apoptotic cells in the remaining viable cells at the end of the experiment (C). In the absence of A2E, VEGFa supplementation increased RPE cell viability in the dark and at 400 nm. VEGFa supplementation of A2E-loaded RPE cells decreased viability at 440 and 630 nm. VEGFa supplementation on RPE cells without A2E did not affect apoptosis upon light exposure. By contrast, VEGFa supplementation on A2E-loaded RPE cells increased apoptosis at 440 nm. Data are expressed as the mean +/- SEM (n = 3). Differences between samples were considered to be significant when p < 0.05 (*), p < 0.01 (**) or p < 0.001 (***).

VEGFa is neuroprotective for different retinal cells [49, 50]. It seemed thus possible that the high content of VEGFa in the cell culture medium could act as a pro-survival factor in A2E loaded cells. Therefore, we tested this possibility by incubating A2E-loaded RPE cells with 10 ng/mL recombinant VEGFa protein for 2 h before and during 18 h of light exposure. This concentration of VEGFa was used based on the VEGF concentrations measured by ELISA in the RPE cell culture medium (Fig 2) to have the same initial VEGF protein concentration in the medium for all experimental conditions. This concentration was also found to be neuroprotective in a previous study on retinal ganglion cells [49]. We assessed viability and apoptosis with the Apolive Glo Assay^TM^ (Promega) 6 h after light exposure. In the absence of A2E, VEGFa only significantly increased RPE cell viability at 400 nm but there was no detectable effect on apoptosis (Fig 3). In the absence of added VEGFa, blue light (400, 430,or 440 nm) decreased cell viability and induced up to a 5.5-fold increase in apoptosis/viability of A2E-loaded RPE cells relative to dark controls (Fig 3), as previously described by our group [45]. Addition of VEGF to the incubation medium resulted in a statistically significant enhancement in the loss of cell viability at 440 and 630 nm in A2E-loaded RPE cells (Fig 3A). This decrease in cell viability correlated with an increase in apoptosis at 440 nm (Fig 3B), which was significant after normalization to the rate of viability (Fig 3C). Differences in VEGF sensitivity observed between 430 and 440 nm could be related to the different ranges of toxicity at these two wavelengths. Indeed, in the absence of VEGF, 430 nm was already much more toxic than 440 nm on A2E-loaded RPE cells and thus could prohibit a further increase in toxicity upon addition of VEGF. VEGF was not protective to A2E-loaded RPE cells exposed to light but further increased light-induced toxicity at 440 nm.

**Fig 4:**
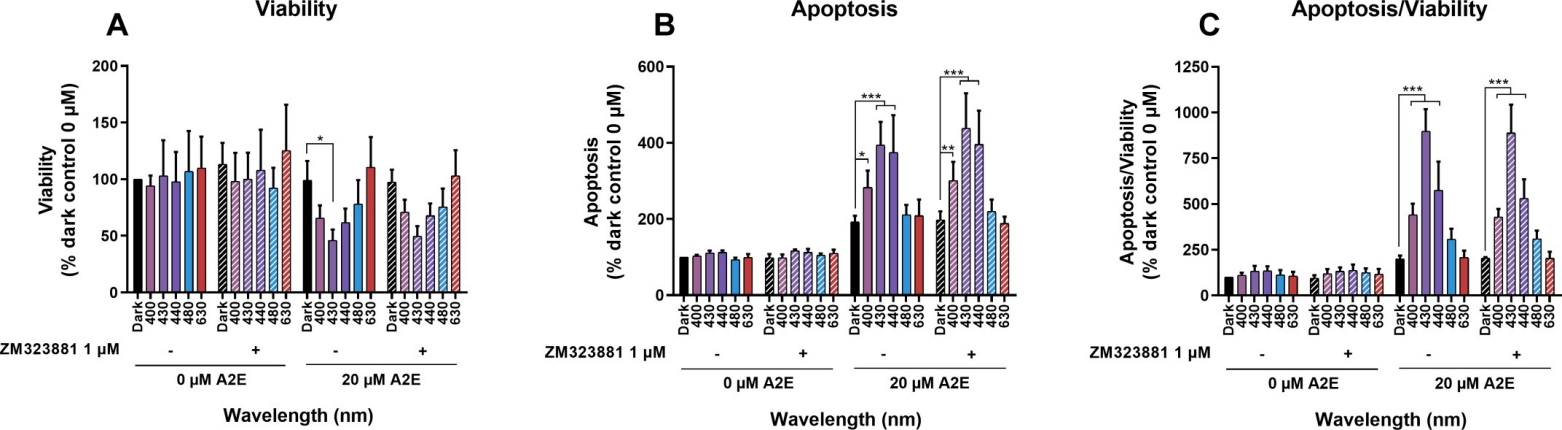
Effects of VEGFR2 inhibition on viability and apoptosis of A2E-loaded RPE cells exposed to light. RPE cells were loaded with 0 or 20 μM A2E and treated with ZM323881 hydrochloride (1 μM), a selective inhibitor of VEGFR2 activity, before being exposed to light for 18 h light exposure. Viability (A) and apoptosis (B) were evaluated 6 h after the end of light exposure with the Apolive Glo AssayTM. The number of apoptotic cells was normalized to cell viability to define the density of apoptotic cells in the remaining viable cells at the end of the experiment (C). VEGFR2 inhibitor did not modify RPE cell viability or apoptosis under any conditions tested. Data are expressed as the mean +/- SEM (n = 4). Differences between samples were considered to be significant when p < 0.05 (*), p < 0.01 (**) or p < 0.001 (***).

A2E-loaded RPE cells synthesized and released VEGF into the extracellular medium at a concentration that enhanced blue-light toxicity at 440 nm (Fig 3). We investigated whether this secreted VEGF contributes to A2E-induced phototoxicity by measuring cell viability and apoptosis in the presence of a selective inhibitor of VEGFR2 (ZM323881 hydrochloride) to block the main exogenous VEGFa signaling pathway in RPE cells [51]. A VEGFR inhibitor concentration of 1 μM was chosen to exert selective efficacy on VEGFR2, while avoiding interaction with VEGFR1, PDGFRb, FGFR1, EGFR, or erbB2, described at higher concentrations [51, 52]. Thus, the VEGFR1 inhibitor can block any autocrine and paracrine effect of VEGFa release by RPE cells in the culture medium. RPE cells were loaded with A2E at 0 or 20 μM, washed, and then incubated with the inhibitor in fresh medium just before starting the 18-h light exposure. We assessed viability and apoptosis 6 h after light exposure (Fig 4A, 4B and 4C). Addition of the inhibitor did not modify RPE cell viability or apoptosis under the experimental conditions tested, consistent with the low VEGFR2 expression in RPE cells (Fig 1). Under our experimental conditions, this autocrine pathway of VEGFa mediated VEGFR2 activation was not critical in inducing A2E-loaded RPE cell degeneration under light exposure.

## Discussion

VEGF plays an important role in the development of the wet form of age-related macular degeneration. Indeed, excessive release of this factor by RPE cells induces rapid neovascularization under the retina. The growth of abnormal vessels in the subretinal space, with a porous wall, causes disruption of the epithelial barrier and leakage of fluids under the retina, which is partially responsible for vision loss. Among the various structures of the eye, the retina and, more specifically RPE cells contain the largest amounts of VEGF protein [47]. Indeed, RPE cells are responsible for the production and secretion of several growth factors, including VEGF, required for all other structures of the eye. Despite their high VEGF content, RPE cells express only low levels of the VEGF receptors, VEGFR1 and VEGFR2, with expression of VEGFR2 being less than that of VEGFR1 [47]. We confirmed the high levels of VEGF mRNA expression and protein synthesis, whereas the level of VEGFR1 mRNA was low and that of VEGFR2 barely detectable. We showed that A2E increased VEGF mRNA levels and VEGF protein synthesis. This result, obtained with primary porcine RPE cells, is consistent with those reported for the ARPE-19 cell line [26, 48]. This A2E-induced increase in VEGF synthesis was attributed to the sustained activation of retinoic acid receptors (RAR) in both the ARPE-19 cell line and Y79 retinoblastoma cells and *in vivo* [25, 26, 53]. Our results are in accordance with these studies, showing that incubation of RPE cells with A2E in the dark increased VEGF synthesis and secretion following a dose-dependent increase in mRNA expression. Our results confirm the A2E-induced potentiation of VEGF synthesis and release, suggesting that A2E accumulation could thus stimulate the neovascularization observed in wet AMD.

Light, more specifically blue light, has been shown to be a cumulative risk factor for the development of AMD [2]. We are all exposed to this environmental factor in a cumulative manner during our lifetime. It is thus of great importance to understand how it can influence the development of AMD in particular the VEGF-related complications of the wet form of this disease. VEGF expression was reported to increase in several studies in which oxidative stress was induced in ARPE-19 cells by the addition of chemical compounds, such as H_2_O_2_ or ethanol [54, 55]. Light-elicited oxidative stress especially in the presence of A2E, may thus also increase VEGF expression. High H_2_O_2_ production has indeed been demonstrated in A2E-loaded cells upon blue light exposure [46, 56]. Furthermore, this light-elicited oxidative stress was found to induce a translocation of mitochondria to the perinuclear area [46, 56], which has been shown to enhance the expression of hypoxia-sensitive genes, such as VEGF, in other cell systems [57]. Others have already reported that bright white light reduces cell survival but increases VEGF synthesis in human primary RPE cells [43]. However, the A2E content in these cells is unknown. Also, an un-tinted intraocular lens (IOL) produced a greater increase in VEGF expression than a blue-light filtering IOL *in vitro* showing that blue light further increases VEGF synthesis [42]. Similarly, white light increased VEGF synthesis in A2E-loaded ARPE-19 cells and this effect was attenuated by a blue-light filtering IOL, suggesting potentiation of VEGF synthesis by blue light stimulation [40, 58]. Surprisingly, we obtained the opposite results; blue light (400, 440, or 480 nm) reduced VEGF mRNA expression and protein synthesis of A2E-loaded primary porcine RPE cells. This difference may be due to the different cell types used in the studies: the ARPE-19 cell line or primary human or porcine RPE cells. The human primary RPE cells were not incubated with A2E, in contrast to our experimental conditions and those under which the ARPE-19 cell line was used [40, 58]. The main difference may indeed lie in the light exposure conditions, because the other groups mainly used bright white light and blue light filtering-IOLs to assess the specific effect of blue light [40, 58]. In contrast, we used specific exposure to 10 nm bandwidths, such that it would be difficult to anticipate the summed response of a white light spectrum. In addition, we did not apply UV light, in contrast to other studies that used white light, possibly demonstrating an effect of UV-absorbing IOL [40]. Several studies on A2E-loaded ARPE-19 cells also restricted their illumination to a specific bandwidth (430 nm), but the illumination periods were very short (3–7 min) in most cases [41], whereas we used moderate irradiance and long-duration illumination (15-18h). Our irradiance levels were indeed moderate (below 1.5 mW/cm^2^) and lower than those of other studies (8–10 mW/cm^2^) [40, 41]. Finally, we used wavelength irradiances that were normalized to the sunlight spectrum that reaches the retina for each wavelength band to mimic the *in vivo* conditions of the natural protection of the crystalline lens as closely as possible. Although blue light has been considered to further enhance the risk of vascular complications in other cell models, our study suggests that it may instead reduce VEGF synthesis and release by A2E-loaded RPE cells when exposed to moderate irradiance normalized to the sunlight spectrum with a long duration exposure.

VEGF has been described to act as an autocrine survival factor in an RPE cell line (APRE-19) under H_2_O_2_-induced oxidative stress via the autocrine VEGFR2 activation pathway [59]. Here, we observed a two-fold increase in VEGF synthesis upon incubation with A2E, as have others [26]. Because blue light (415–455 nm) exposure induces substantial H_2_O_2_ production in A2E-loaded RPE cells leading to RPE cell apoptosis [45, 46, 56], we examined whether VEGF can act as a survival factor for A2E-loaded cells exposed to blue light. Surprisingly, addition of VEGF to the culture medium (10 ng/mL) increased blue-light toxicity at 440 nm in A2E-loaded RPE cells. These results are consistent with the *in vivo* prevention of light damage to the RPE by anti-VEGF strategies [44]. VEGFa binds to both VEGF receptors VEGFR1 (Flt-1) and VEGFR2 (KDR/Flk-1). VEGFR2 appears to mediate almost all intracellular signaling pathways in the vascular endothelium [6, 7]. However, we showed that RPE cells exhibit much lower levels of VEGFR2 mRNA than VEGFR1 mRNA. VEGFR1 mRNA levels were substantially increased by blue light (440 nm) exposure in A2E-loaded RPE cells. Although, a VEGFR2 inhibitor did not show any effect on cell survival due to the secreted VEGF in our culture medium, it does not exclude that the VEGFR2 signaling pathway is responsible for the VEGF enhancement of blue light toxicity on A2E-loaded RPE cells. In AMD, the accumulation of A2E may thus lead to VEGF synthesis and release by RPE cells, such that the VEGF released into the extracellular medium may not only induce angiogenesis but also increase blue light toxicity. As a consequence, VEGF therapy could thus prevent angiogenesis and protect RPE cells from light damage. Interestingly, blue light may naturally mimic such anti-VEGF therapy in RPE cells by increasing VEGFR1 expression and decreasing VEGF expression. VEGFR1 can indeed trap free VEGF as do the therapeutic molecules in all anti-VEGF therapies [60, 61]. However, this anti-angiogenic effect acts in parallel with the very high risk of blue-light damage.

## Conclusion

Anti-VEGF therapies have become a major treatment modality in the daily care of wet AMD and diabetic retinopathy to suppress the growth of neovessels and/or macular edema [5]. The photosensitizer A2E can upregulate both VEGF mRNA and protein levels in RPE cells by activating retinoic receptors [26]. A2E photosensitization by blue light induce oxidative stress and RPE cell death in an *in vitro* model of AMD [45, 46]. Here, we showed that blue light exposure of A2E-loaded RPE cells for 15 h at moderate irradiance, normalized to sunlight irradiance reaching the retina, can also down regulate VEGF synthesis while up-regulating VEGFR1 mRNA expression. The upregulation of VEGFR1 may act as a VEGF-trap or as a protective mechanism against oxidative stress [60–62]. Surprisingly, we found that supplementation with VEGF, a well-known pro-survival factor, can further increase blue light toxicity to A2E-loaded RPE cells. The conversion of VEGF pro-survival signaling into cell apoptosis has already been described in endothelial cells [63, 64]. This suggests that anti-VEGF therapy may not only resolve vascular complications, but may also limit the VEGF-elicited enhancement of blue-violet light toxicity on A2E-loaded RPE cells.
